# Positive classroom climate buffers against increases in loneliness arising from shyness, rejection sensitivity and emotional reactivity

**DOI:** 10.3389/fpsyt.2023.1081989

**Published:** 2023-03-23

**Authors:** Gintautas Katulis, Goda Kaniušonytė, Brett Laursen

**Affiliations:** ^1^Institute of Psychology, Mykolas Romeris University, Vilnius, Lithuania; ^2^Department of Psychology, Florida Atlantic University, Fort Lauderdale, FL, United States

**Keywords:** loneliness, classroom climate, shyness, rejection sensitivity, emotional reactivity

## Abstract

Loneliness is detrimental to well-being, particularly during the transition into and early years of adolescence when peer relations are ascendant. Shy and emotionally sensitive youth, who often spend considerable time alone, have known vulnerabilities to loneliness. Studies of young children suggest that a supportive classroom context may mitigate adjustment risks, reducing victimization and improving a sense of belonging. Herein we extend this work to older students, testing the hypothesis that a positive classroom climate protects temperamentally vulnerable children (i.e., those who are shy, emotionally reactive, or sensitive to rejection) from escalating levels of loneliness across the course of a school year. A community sample of 540 (277 boys, 263 girls) Lithuanian students in grades 5–7 (10–14 years old) completed identical surveys twice, 4–5 months apart. Self-reports assessed shyness, emotional reactivity, and rejection sensitivity, as well as perceived positive classroom climate and loneliness. Path analyses indicated that longitudinal associations from shyness, emotional reactivity, and rejection sensitivity to increased loneliness were mitigated by positive classroom climate. In each case, temperamental vulnerability anticipated greater loneliness for youth reporting low but not high positive classroom climate. The results held after accounting for several potential confounding variables. The findings have practical implications, suggesting that scholars and practitioners redouble efforts to improve classroom support, particularly for temperamentally vulnerable children who are at elevated risk for solitude, loneliness, and attendant mental health challenges.

## 1. Introduction

Youth are at special risk for loneliness during late childhood and early adolescence, when peer relations are ascendent (1) The debilitating nature of loneliness cannot be overstated: Lonely children and adolescents present a host of short- and long-term mental health problems (2). Not everyone is equally disposed to being lonely. Shy, sensitive to rejection, and emotionally reactive youth have temperamental vulnerabilities that place them at heightened risk for loneliness (3–5). Alert to these risks, investigators have focused on factors that might mitigate loneliness in vulnerable youth. Chief among them is classroom climate, which is known to buffer against adjustment difficulties among children with peer difficulties (6). Using a community sample of Lithuanian youth ages 10 to14, we test the hypotheses that perceived positive classroom climate moderates longitudinal associations from temperamental vulnerabilities (i.e., shyness, emotional reactivity, and rejection sensitivity) to heightened loneliness.

Loneliness is a painful state of unwanted social isolation, often occurring in response to perceived relationship deficits (7, 8). Lonely children report higher rates of solitude and preference for solitude than their nonlonely counterparts (9), not because they enjoy being alone but rather typically to avoid social discomfort (10). Developmental changes conspire to make the transition into adolescence a period of heightened loneliness (1). By some estimates, up to 30% of youth report regularly feeling lonely or very lonely (11). Buffeted by uncertainties about identity, improved perspective taking abilities, and expectations for autonomy, opportunities for social isolation multiply as the social world is transformed from one constructed by adults to one dominated by peers. Lonely youth suffer, particularly from victimization and depression (12, 13).

Some youth have temperamental vulnerabilities that heighten their risk for loneliness. We focus here on children who present elevated levels of shyness, rejection sensitivity, or emotional reactivity, three risk factors with biological origins. *Shyness* is characterized by conflicting motivations to engage and avoid peers; interactions with agemates are appealing but concerns about negative evaluations can prompt distress and a desire to withdraw (14). *Rejection sensitivity* is a heightened tendency to “anxiously expect, readily perceive, and overreact” to social rejection (15). *Emotional reactivity* describes a heightened (often negative) emotional response to affective situations (16). Grounded in temperament, each manifests early in life and is relatively stable across development (17–19). Although modestly correlated [*r*s range from.300 to.450 (20–22)], the constructs are conceptually and empirically distinct. Shyness stems from a fear of novelty (17), rejection sensitivity has biopsychosocial origins associated with sensitive interpretation of ambiguous early social experiences (23), and emotional reactivity reflects a low emotional threshold for external stimuli (19).

Shy, reactive, and sensitive to rejection youth are assumed to be prone to loneliness because of two underlying mechanisms: An inclination to interpret social situations negatively and social inhibitions that interfere with the creation and maintenance of social ties (3, 24, 25). Solitude may be preferred to the uncertainties and potential pain of social engagement (26, 27), which heightens risks for loneliness (28). Many temperamentally vulnerable youth possess off-putting characteristics, a problem exacerbated by cascading social skills deficits brought on by minimal peer contact (29). A self-fulfilling prophecy unfolds whereby hypervigilance prompts negative interpretations or inaccurate interpretations of social cues and inappropriate emotional reactions to social situations, alienating peers who avoid them as unattractive interaction partners, forcing isolation and fostering loneliness. (30). Finally, temperamentally vulnerable children may find themselves with few friend options aside from other interpersonally challenged agemates, who may be equally unsatisfying partners (31, 32).

Consistent with the above, research indicates that temperamentally vulnerable children are at heightened risk for loneliness. The evidence for shyness is particularly compelling. Shy youth report feeling lonely more frequently than those who are not shy (5). Rejection sensitivity has also been linked to longitudinal increases in adolescent loneliness (4). Less is known about emotional reactivity. Longitudinal links between parent-reported negative reactivity and related constructs have been established. Specifically, among adolescents, emotional intelligence has been linked with loneliness (33) and emotional reactivity has been linked to emotional problems (34).

Our study starts from the premise that perceived support from classmates can help protect temperamentally vulnerable children against loneliness. Research based on social information processing mechanisms [predisposed emotional responses to social cues based on past experiences which impact the interpretation of the situation and the following behaviors] suggests that shy youth are less prone to interpret social situations in a self-defeating manner when interacting with supportive friends (35). Perceiving the classroom as supportive is the postulated mechanism through which the risk of loneliness is reduced. In supportive classes, vulnerable children may interpret social situations as pleasant, lowering the risk of loneliness as children reframe social experiences (36). Additionally, supportive classrooms have higher group cohesion and mutual respect, avoiding situations that elicit mismatched emotional responses that can lead to social exclusion (37). As a consequence, temperamentally vulnerable youth may feel comfortable engaging in interactions that lead to meaningful social connections (38). Additional social opportunities, in turn, help youth improve social skills in ways that diminish the tendency to withdraw (39).

Our study is novel in that we focus on perceptions of classroom climate as an index of support. Positive classroom climate is defined as the perceived tenor of the classroom and the degree to which students feel comfortable and at ease in the classroom and with classmates (40). Perceived classroom climate has been shown to moderate concurrent association between other biologically-linked traits (e.g., effortful control) and depressive symptoms (41). Longitudinal data from college students (42) and concurrent data from young adolescents (3) agree that friend support protects youth against loneliness arising from peer difficulties. Supportive classrooms should operate in a similar manner. For instance, two studies of indicate that school and classroom climate buffered against loneliness among victimized children and young adolescents (43, 44). Similarly, a 3-year study of primary school students indicated that anxious withdrawn children were less likely to be excluded in supportive classrooms than in unsupportive classrooms (37).

The present study utilizes a community sample of Lithuanian primary and middle school students to examine whether perceived positive classroom climate moderates longitudinal associations from shyness, emotional regulation, and rejection sensitivity to increases in loneliness across a four-month period during a single school year. We hypothesized that shyness, rejection sensitivity and emotional reactivity would predict increases in loneliness for youth who perceived low but not high levels of classroom support. Given that temperamental vulnerabilities may have different social outcomes depending on gender (e.g., shy girls face less rejection than shy boys) (45), we compared boys and girls on patterns of association. Because loneliness is associated with emotional problems (34) and the number and quality of friendships (41), each was included as a covariate in supplemental analyses. Victimization has been linked to perceived classroom climate (46) as well as shyness (47), emotional reactivity (48), and rejection sensitivity (49), so peer reports of relational and physical victimization were also included as potential confounders.

## 2. Methods

### 2.1. Participants

Participants included 540 students in 5th (97 boys, 89 girls; *M_age_* = 10.85, *SD_age_* = 0.410), 6th (84 boys, 81 girls; *M_age_* = 11.83, *SD_age_* = 0.437), and 7th (96 boys, 93 girls; *M_age_* = 12.73, *SD_age_* = 0.457) grades. Nearly all participants were of Lithuanian ethnicity. Most lived with two biological parents (69.3%); the remainder lived in blended (13.7%) or single parent (15.6%) households, or with guardians or grandparents (1.5%). Approximately 9.5% received free meals at school.

### 2.2. Procedure

All 5-7th graders (attending 33 classrooms in 4 middle schools) in the community were invited to participate. Written parent consent and student assent were required for participation. Trained research assistants administered questionnaires in classes on computer tablets in September 2021 and February 2022. The study was approved by the university ethics committee (Nr. 6/202).

The initial participation rate was 65.2%. Of the 540 students who participated at Time 1, 525 also participated at Time 2. There were no differences in any study or demographic variables between students who did and did not participate at both time points. Item-level missingness ranged from 3.3–22.6% (*M* = 11.7%, *SD* = 6.0). Little’s MCAR test indicated that data were missing completely at random, *χ*^2^(16,101) = 15,689.330, *p* = 0.990. Item-level missing data were imputed with an EM algorithm with 25 iterations. Missing wave-level data were handled with FIML.

### 2.3. Measures

Participants completed the same surveys at both time points. Unless otherwise indicated, items were rated on a scale ranging from 1 (*strongly disagree*) to 5 (*Strongly agree*). Scores were averaged. Higher scores indicated greater levels of a variable. Internal reliabilities are presented in Table 1. All items for each variable are listed in Supplementary Table S1.

**Table 1:** Interclass correlations, means, and standard deviations.

Variable	1	2	3	4	5	*M* (SD)	*Cronbach’s a*
1. Loneliness	543** [0.451, 0.624]	571** [0.494, 0.642]	0.453** [0.381, 0.515]	0.518** [0.434, 0.601]	−0.520** [−0.600, −0.434]	1.854 (0.982)	0.950
2. Shyness	0.589** [0.511, 0.654]	0.602** [0.528, 0.666]	0.385** [0.299, 0.462]	0.455** [0.373, 0.538]	−0.342** [−0.433, −0.351]	2.174 (1.032)	0.858
3. Emotional reactivity	0.424** [0.339, 0.495]	0.343** [0.249, 0.425]	0.543** [0.474, 0.607]	0.418** [0.343, 0.490]	−0.215** [−0.308, −0.111]	3.070 (0.869)	0.836
4. Rejection sensitivity	0.478** [0.390, 0.555]	0.444** [0.354, 0.517]	0.367** [0.287, 0.439]	0.574** [0.495, 0.648]	−0.357** [−0.455, −0.258]	6.902 (4.536)	0.730
5. Perceived positive classroom climate	−0.569** [−0.637, −0.483]	−0.402** [−0.489, −0.313]	−0.185** [−0.270, −0.079]	−0.357** [−0.443, −0.262]	0.628** [0.556, 0.695]	3.717 (0.879)	0.848
*M (SD*)	1.858 (0.994)	2.251 (1.014)	3.121 (0.845)	6.937 (4.449)	3.768 (0.8613)		
							*Cronbach’s a*	0.941	0.826	0.808	0.672	0.795		

*N* = 540. Time 1 concurrent correlations are shown below the diagonal. Time 2 concurrent correlations are shown above the diagonal. Autocorrelations are presented on the diagonal. 95% confidence intervals in brackets.

*p < 0.05; **p < 0.01.

#### 2.3.1. Shyness

Participants completed a 3-item shyness scale from the Motivations for Withdrawal Questionnaire (14) (e.g., “I am shy”).

#### 2.3.2. Perceived positive classroom climate

Participants completed a 3-item positive classroom climate scale adapted from the Peer Context Questionnaire (40) (e.g., “In this class, I feel comfortable”).

#### 2.3.3. Emotional reactivity

Participants completed a 5-item adapted version of emotional reactivity scale (50) (e.g., “My feelings get hurt easily”).

#### 2.3.4. Rejection sensitivity

Participants completed an abbreviated 6-item rejection sensitivity scale adapted from the Rejection Sensitivity Questionnaire (51) (e.g., “How nervous would you feel about whether anyone will choose you?”). Items were rated from 1 (*not worried at all*) to 5 (*very worried*). For each of 3 hypothetical social situations, responses to 2 questions were multiplied, then averaged.

#### 2.3.5. Loneliness

Participants completed a 3-item adapted version of loneliness scale (52) (e.g., “I feel alone at school”).

#### 2.3.6. Potential confounding variables

To isolate effects to the main study variables, supplemental analyses were conducted that included, as Time 1 covariates and Time 2 predictors, variables known to correlate with loneliness, shyness, emotional reactivity, and/or rejection sensitivity. *Emotional problems*, previously linked to shyness and loneliness (53, 54), were measured with 6 items from the Strengths and Difficulties Questionnaire (55) (e.g., “I worry a lot”). Additionally, participants completed a peer assessment questionnaire consisting of a roster on which they identified classmates who best fit a description (56). Unlimited same and other sex nominations were permitted. Nominations received were summed and standardized within classes (57). Two measures of peer liking were included, previously linked to rejection sensitivity (58): (a) *rejection* (“someone you do not like to spend time with”) and (b) *acceptance* (“someone you like to spend time with”). Two measures of peer victimization were included, previously linked to loneliness (59). (c) *relational victimization* (“Someone who is called names or teased by others”) and (d) *physical victimization* (“someone who is hit or pushed by others”). Finally, the quantity and quality of friendships were assessed, previously linked to loneliness (6) and rejection sensitivity (60). Participants identified up to 5 friends, from which the *number of reciprocated friendships* (*M* = 1.90, *SD* = 1.52) was determined. For the first and second best friends, each participant completed an abbreviated version of the Network of Relationships Inventory (61), with 5 items describing *friendship social support* (e.g., “My friend and I help each other out”) and 4 items describing *friendship negativity* (e.g., “My friend and I argue with each other”). Scores for the two best friends were averaged.

### 2.4. Plan of analysis

**Figure 1:**
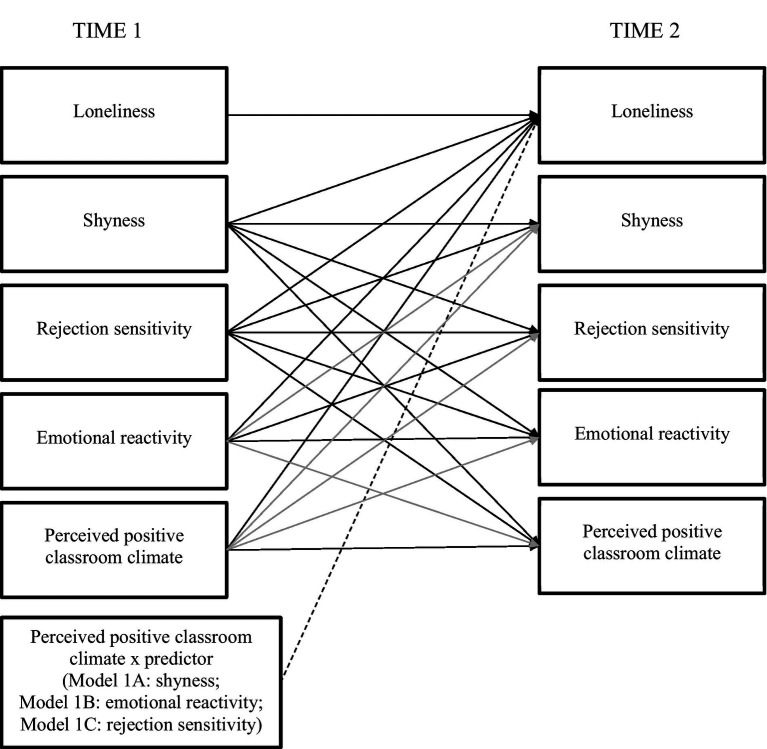
Longitudinal associations from shyness, rejection sensitivity, and emotional reactivity to loneliness: Direct and moderated analytic models. Gray lines represent nonsignificant paths that were trimmed from the final models. Solid lines represent paths that were included in Model 0 and Model 1; the dashed line represents one of three moderator variable paths that were separately included in Models 1A, 1B, and 1C. Concurrent correlations (not depicted) are given in Table 1.

Analyses tested the hypothesis that perceived positive classroom climate moderates longitudinal associations from shyness, emotional reactivity, and rejection sensitivity to changes in adolescent loneliness. A two-step procedure for estimating moderated paths was conducted in Mplus 8.4. Figure 1 illustrates the analytic model. The model is akin to a residual change model, such that autoregressive effects represent the stability of a variable. By accounting for stability and within time correlations, cross-lagged paths predict residual change. The COMPLEX function was applied to address potential classroom-level differences; the same pattern of statistically significant results emerged without it, implying minimal variation across classes (56). Intraclass correlations between the main variables, calculated within classrooms, ranged from 0.004 to 0.043 suggesting that classroom nesting accounted for little to no variability (62). Standard model fit indices were applied (63). The chi-squared index should be nonsignificant; the root-mean-square error of approximation (RMSEA) should be 0.06 or lower; the Tucker-Lewis index (TLI) should be greater than 0.95 (64).

In the first step, a model (Model 0) without any interaction terms was estimated. Model 0 was trimmed by removing nonsignificant cross-lagged paths, provided that doing so did not worsen model fit (64).

In the second step, interaction terms (all measured at Time 1) were added to the model (Model 1). Three moderated models were tested in separate analyses: (1) Shyness predicting changes in loneliness moderated by perceived positive classroom climate (Model 1A); (2) Emotional reactivity predicting changes in loneliness moderated by perceived positive classroom climate (Model 1B); and (3) Rejection sensitivity predicting changes in loneliness moderated by perceived positive classroom climate (Model 1C).

In all models, grade in school was included as a Time 1 covariate to account for mean level differences (see below); the same pattern of statistically significant results emerged when grade was omitted.

Follow-up simple slope analyses probed statistically significant moderated associations at high (1 *SD* above the mean) and low (1 *SD* below the mean) levels of the moderator. The procedure estimates slopes at given levels of the predictor, utilizing the entire sample (65).

## 3. Results

### 3.1. Preliminary analysis

Table 1 presents interclass correlations (Pearson’s *r*). At both time points, shyness, rejection sensitivity, emotional reactivity and loneliness were positively correlated with each other and negatively correlated with perceived positive classroom climate.

Separate 2 (gender) × 3 (grade) ANOVAs were conducted for each study variable, with time as a repeated measure. Statistically significant (*p* < 0.05) main effects of gender emerged for shyness [F (1)=21.055, *d* = 0.402], emotional reactivity [F (1)=71.757, *d* = 0.742], rejection sensitivity [F (1)=27.160, *d* = 0.454], and loneliness [F (1)=18.104, *d* = 0.375]. In each case, girls scored higher than boys. Statistically significant gender [F (1)=12.212, *d* = 0.306] and time [F (1)=4.262, *d* = 0.179] main effects on perceived positive classroom climate were qualified by a gender x time interaction [*F* (1)=5.030, *p* = 0.025]. Follow-up t-tests revealed that boys reported decreases in perceived positive classroom climate [F (1)=10.196, *d* = 0.392], whereas girls did not [F (1)=0.027, *d* = 0.000]. There was a grade x time interaction for loneliness [*F* (2)=3.676, *p* = 0.026]. Loneliness increased among 7th graders [F (1)=4.945, *d* = 0.326], but not among 5th [F (1)=2.749, *d* = 0.246] or 6th [F (1)=0.004, *d* = 0.001] graders.

### 3.2. Longitudinal associations from initial shyness, rejection sensitivity, and emotional reactivity to subsequent loneliness moderated by initial perceived positive classroom climate

#### 3.2.1. Shyness, rejection sensitivity, and emotional reactivity predicting changes In loneliness

3.2.1 A trimmed version of Model 0 (without interaction terms) fit the data. Five nonsignificant paths were trimmed from the model: Time 1 perceived positive classroom comfort predicting Time 2 shyness (β = 0.029), emotional reactivity (β = −0.044) and rejection sensitivity (β = −0.042); and Time 1 emotional reactivity predicting Time 2 shyness (β = 0.060) and perceived positive classroom climate (β = −0.017). Results are presented in Table 2.

**Table 2:** Longitudinal associations from Time1 shyness, emotional reactivity, rejection sensitivity to Time 2 loneliness moderated by Time 1 perceived classroom climate: Results from path analysis.

Longitudinal path	β	CI [95%]	*p*
*Cross lagged paths (Model 0)*			
T1 Emotional reactivity → T2 Loneliness	0.093**	[0.024, 0.162]	0.008
T1 Shyness → T2 Loneliness	0.101*	[0.015, 0.187]	0.021
T1 Classroom climate → T2 Loneliness	−0.13**	[−0.203, −0.057]	0.001
T1 Rejection sensitivity → T2 Loneliness	0.125**	[0.042, 0.208]	0.003
T1 Shyness → T2 Classroom climate	−0.077*	[−0.152, −0.001]	0.046
T1 Rejection sensitivity → T2 Classroom climate	−0.127**	[−0.201, −0.052]	0.001
T1 Shyness → T2 Emotional reactivity	0.117**	[0.037, 0.197]	0.004
T1 Rejection sensitivity → T2 Emotional reactivity	0.108*	[0.026, 0.189]	0.010
T1 Rejection sensitivity → T2 Shyness	0.129**	[0.053, 0.205]	0.001
T1 Emotional reactivity → T2 Rejection sensitivity	0.095*	[0.022, 0.168]	0.011
T1 Shyness → T2 Rejection sensitivity	0.145**	[0.068, 0.202]	0.001
*Autoregressive paths (Model 0)*			
T1 Loneliness → T2 Loneliness	0.289**	[0.204, 0.374]	0.000
T1 Shyness → T2 Shyness	0.545**	[0.479, 0.611]	0.000
T1 Rejection sensitivity → T2 Rejection sensitivity	0.479**	[0.407, 0.551]	0.000
T1 Emotional reactivity → T2 Emotional reactivity	0.459**	[0.389, 0.530]	0.000
T1 Classroom climate → T2 Classroom climate	0.554**	[0.491, 0.617]	0.000
*Model 1A*			
T1 Shyness × T1 Classroom climate → T2 Loneliness	−0.364**	[−0.563, −0.166]	0.001
*Model 1B*			
T1 Emotional reactivity × T1 Classroom climate → T2 Loneliness	−0.502**	[−0.784, −0.221]	0.011
*Model 1C*			
T1 Rejection sensitivity × T1 Classroom climate → T2 Loneliness	−0.420**	[−0.614, −0.226]	0.000

*N* = 540. Standardized beta weights reported. Model 0 describes results without interaction terms. Models 1 (ABC) describe results from separate models that included interaction terms. Nonsignificant paths were trimmed. Concurrent correlations are given in Table 1. Model fit the data for model 0 [*χ*^2^(14) = 15.541, *p* = 0.342; TLI = 0.997; RMSEA = 0.014 (0.000, 0.045)], Model 1A [*χ*^2^(18) = 24.890, *p* = 0.127; TLI = 0.987; RMSEA = 0.027 (0.000, 0.050)], Model 1B [*χ*^2^(18) = 26.350, *p* = 0.092; TLI = 0.984; RMSEA = 0.029 (0.000, 0.052), and Model 1C [*χ*^2^(18) = 26.974, *p* = 0.080; TLI = 0.983; RMSEA = 0.003 (0.000, 0.053)]. Classroom climate = Positive perceived classroom climate.

*p < 0.05; **p < 0.01.

We focus first on the longitudinal paths of interest. There were positive associations from Time 1 shyness, Time 1 rejection sensitivity and Time 1 emotional reactivity to Time 2 loneliness. There also was a negative association from Time 1 perceived positive classroom climate to Time 2 loneliness.

Several additional cross-lagged paths were statistically significant. Time 1 rejection sensitivity was negatively associated with Time 2 perceived positive classroom climate, and positively associated with Time 2 emotional reactivity and Time 2 shyness. Time 1 shyness was positively associated with Time 2 emotional reactivity and Time 2 rejection sensitivity, and negatively associated with Time 2 perceived positive classroom climate. Time 1 emotional reactivity was positively associated with Time 2 rejection sensitivity. All stability coefficients were statistically significant.

#### 3.2.2. Shyness predicting changes in loneliness, moderated by perceived positive classroom climate

**Figure 2:**
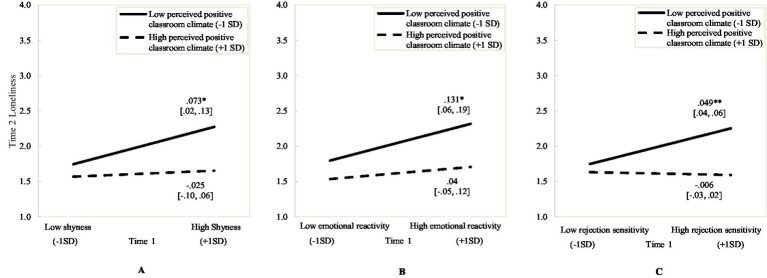
Time 1 Shyness **(A)**, Emotional reactivity **(B)** and Rejection sensitivity **(C)** predicting Time 2 Loneliness at low and high levels of perceived positive classroom climate. *N* = 540; * *p* < 0.05; ** *p* < 0.001.

Model 1A (with the shyness x perceived positive classroom climate interaction term) fit the data. The interaction term predicted changes in loneliness from Time 1 to Time 2. Figure 2 presents results from simple slope analyses. There was a significant positive association from Time 1 shyness to Time 2 loneliness at low (−1 *SD*) but not high (+1 *SD*) levels of perceived positive classroom climate. For youth reporting low perceived positive classroom climate, higher initial shyness was associated with increased loneliness across the school year.

#### 3.2.3. Emotional reactivity predicting changes in loneliness, moderated by perceived positive classroom climate

Model 1B (with the emotional reactivity x perceived positive classroom climate interaction term) fit the data. The interaction term predicted changes in loneliness from Time 1 to Time 2. Figure 2 presents results from simple slope analyses. There was a significant positive association from Time 1 emotional reactivity to Time 2 loneliness at low (−1 *SD*) but not high (+1 *SD*) levels of perceived positive classroom climate. For youth reporting low perceived positive classroom climate, higher initial emotional reactivity was associated with increased loneliness across the school year.

#### 3.2.4. Rejection sensitivity predicting changes in loneliness, moderated by perceived positive classroom climate

Model 1C (with the rejection sensitivity x perceived positive classroom climate interaction term) fit the data. The interaction term predicted changes in loneliness from Time 1 to Time 2. Figure 2 presents results from simple slope analyses. There was a significant positive association from Time 1 rejection sensitivity to Time 2 loneliness at low (−1 *SD*) but not high (+1 *SD*) levels of perceived positive classroom climate. For youth reporting low perceived positive classroom climate, higher initial rejection sensitivity was associated with increased loneliness across the school year.

#### 3.2.5. Supplemental analyses

Additional analyses were conducted to rule out the possibility that associations were driven by unobserved variables previously linked to the predictor, moderator, and dependent variables. With one exception, the same pattern of statistically significant results emerged when potential confounding variables (i.e., gender, self-reports of emotional problems, number of reciprocated friendships, friendship social support, friendship negativity, peer reports of relational victimization and physical victimization, and peer reports of rejection and acceptance) were separately included as Time 1 covariates and as predictors of Time 2 outcomes. When emotional problems were included in the model 0, the Time 1 shyness to Time 2 loneliness path became marginally significant (β = 0.080; *p* = 0.085).

Multiple group contrasts identified only one gender difference in cross-lagged associations (Δ*χ*^2^ = 8.92, *p* = 0.002): Time 1 rejection sensitivity was positively associated with Time 2 loneliness for boys (β = 0.051, *p* = 0.001) but not for girls (β = 0.011, *p* = 0.341).

## 4. Discussion

We followed a community sample of pre- and early adolescents over the course of a single school year to examine the mitigating role of perceived positive school climate in the development of loneliness among temperamentally vulnerable (i.e., shy, sensitive to rejection, or emotionally reactive) children. The results indicated that perceived positive classroom climate moderates longitudinal associations. In each case, temperamental vulnerabilities anticipated greater loneliness for youth reporting low but not high positive classroom climate.

The findings replicate and extend insights into the antecedents of loneliness. In terms of replication, the findings add to the long list of studies indicating that shy children are especially vulnerable to loneliness (5). Others have also reported that rejection sensitivity increases loneliness (25). Far fewer longitudinal studies have examined whether emotionally reactive children are similarly at risk (3). Taken together with results from previous research, our findings underscore the painful and potentially debilitating costs of loneliness that confront emotionally vulnerable children.

We are not the first to find that a positive classroom climate buffers against conditions that might otherwise have an adverse impact on development. Perceptions of classroom support protect against the untoward consequences of victimization (46, 66), and mitigate the effects of low effortful control on conduct problems (67) and depressive symptoms (36). As such, the findings align with social information processing theory (35), which posits that temperamentally vulnerable youth in classrooms perceived as supportive tend to interpret challenging social situations as benign and nonthreatening. Different processes may be at work depending on whether risks for loneliness have origins in overly sensitive perceptions of social situations (3) or relationship difficulties caused by unattractive traits (31, 32). Shy and sensitive youth may perceive supportive classrooms as a safe place where temperamental characteristics are not a social liability, providing confidence to build ties with classmates. Supportive classrooms are characterized by high engagement and positive peer and teacher interactions (68). Teachers and classmates may work to minimize the time the emotionally vulnerable spend alone and avoid activities that exclude or marginalize members. Finally, supportive classrooms are known to embrace prosocial norms (43), which may disrupt the self-fulfilling prophecy cycle among sensitive children or counteract incipient loneliness among youth so inclined.

Classroom climate has a downstream influence on solitude. Start from the premise that perceptions of a positive classroom climate are joined with perceptions of positive peer experiences (69). Children and adolescents who enjoy spending time with classmates may leap at opportunities to spend time together out of class, accepting and making social invitations, and enrolling in clubs and after-school activities. Additional social experiences may provide temperamentally vulnerable children with much needed social skills practice, bolstering confidence in abilities and reducing withdrawal tendencies (39, 70). Unsupportive classrooms, in contrast, may increase the likelihood that shy and emotionally sensitive children seek to be alone when out of school (37). Discouraged by interpersonal missteps and fearful of replicating painful peer experiences, temperamentally vulnerable children may learn that solitude is safer and preferable (27). In the process, children who most need the company of others instead fall further behind in social skills, developing a (sometimes well-deserved) reputation for awkwardness, which can make successful social integration more difficult in the future.

Other replicated results should bolster confidence in our novel findings. Consistent with previous reports (21, 71), shyness, emotional reactivity, and rejection sensitivity were interrelated longitudinally, such that higher levels of one begat increases in another. We also found that shy and rejection sensitive children were least likely to report that classroom climate improved over the course of the school year, recalling findings from other studies in which less empathetic children (who are lower in emotion regulation) and children with more behavioral problems perceived declining levels of school climate (72, 73).

Our study is not without limitations. First, reliance on self-report variables increases the risk of bias arising from shared-reporter variance. This problem lacks an easy solution, however, because many of the variables of interest focus on child feelings and perceptions, which are not reliably gaged by teachers or parents (74) and whose impact may vary as a function of the discrepancy between actual and ideal self-perceptions (75). Observational data could help distinguish between the impact of perceived and actual classroom climate. Second, it was not possible, with two waves of data, to conduct a random intercept model. In its absence, conclusions about within-individual cross-lagged associations must be tempered, because changes in loneliness may be a product of between-person effects (76).Person-oriented analyses may be better suited to identifying constellations of interpersonal and individual factors tied to adaptive and maladaptive outcomes (77). Third, perceived positive classroom climate is tied to the child’s perceptions of relationships with friends and teachers (78, 79). The same pattern of results emerged when we controlled for friendship quality, suggesting that classroom climate captures more than just getting along with friends. Unfortunately, we lacked data on teacher-child relationships and so cannot make conclusions about the degree to which climate is distinct from getting along with teachers. Finally, the participants lived in a small, homogeneous Northern European community. Those unfamiliar with Lithuania may be hesitant to generalize from its populace. Once involuntarily situated in the Soviet Union, Lithuania is currently a member of the European Union. Students in Lithuania resemble those in other Western European nations with regard to adolescent norms, values, and development (80). Of course, it remains to be seen whether findings from this sample generalize to other, dissimilar contexts.

The results emphasize the importance of perceptions of classroom climate for the well-being of temperamentally vulnerable youth. Future research could investigate the processes through which positive classroom climate protects vulnerable adolescents (67) or classroom level characteristics which determine it being perceived as positive by vulnerable youth (37). This research has implications for classroom oriented interventions that extend well beyond loneliness, with the potential to change the lives of many youths who might otherwise develop a snowballing cascade of interpersonal and mental health challenges.

## Data availability statement

The raw data supporting the conclusions of this article will be made available by the authors, without undue reservation.

## Ethics statement

The studies involving human participants were reviewed and approved by Mykolas Romeris University, Institute of Psychology, Committee of Psychological Research Ethics. DECISION Nr. 6/−202. Written informed consent to participate in this study was provided by the participants’ legal guardian/next of kin.

## Author contributions

All authors listed have made a substantial, direct, and intellectual contribution to the work, and approved it for publication.

## Funding

This project has received funding from European Social Fund (project no 09.3.3-LMT-K-712-17-0009) under grant agreement with the Research Council of Lithuania (LMTLT).

## Conflict of interest

The authors declare that the research was conducted in the absence of any commercial or financial relationships that could be construed as a potential conflict of interest.

## Publisher’s note

All claims expressed in this article are solely those of the authors and do not necessarily represent those of their affiliated organizations, or those of the publisher, the editors and the reviewers. Any product that may be evaluated in this article, or claim that may be made by its manufacturer, is not guaranteed or endorsed by the publisher.

## Supplementary material

The Supplementary material for this article can be found online at: https://www.frontiersin.org/articles/10.3389/fpsyt.2023.1081989/full#supplementary-material
