# Supplementary material for: Positive classroom climate buffers against increases in loneliness arising from shyness, rejection sensitivity and emotional reactivity
Source: Front Psychiatry. 2023 Mar 23;14:1081989. doi: 10.3389/fpsyt.2023.1081989 (PMC10076792; doi:10.3389/fpsyt.2023.1081989)
Supplement: Supplementary file 1 [file Table_1.DOCX]

Supplementary Material

# Supplementary Tables

**Table S1**. Questionnaire items

|  | **Emotional problems** *(α=0.770–0.893.)* |
| --- | --- |
| **Q1** | I get a lot of headaches or stomachaches |
| **Q2** | I worry a lot. |
| **Q3** | I am often unhappy or sad or empty |
| **Q4** | I have many fears, I am easily scared |
| **Q5** | I am nervous in new situations. I easily lose confidence (wording in Lithuanian: New environment frightens me) |
| **Q6** | I am often unhappy |
|  | **Loneliness** *(α=.941-.950).* |
| **Q1** | I feel alone at school. |
| **Q2** | I feel left out of things at school. |
| **Q3** | I’m lonely at school. |
|  | **Shyness** *(α=.826-.858).* |
| **Q1** | I am shy. |
| **Q2** | I want to play with other kids but I don’t because I’m afraid. |
| **Q3** | I spend time alone because I’m too timid and shy. |
|  | **Emotional reactivity** *(α=.808-.836).* |
| **Q1** | My feelings get hurt easily. |
| **Q2** | I tend to get very emotional very easily. |
| **Q3** | I am often bothered by things that other people don’t react to. |
| **Q4** | My emotions go from neutral to extreme in an instant. |
| **Q5** | My moods change very quickly. |
|  | **Perceived Positive Classroom climate** *(α=.795-.848).* |
| **Q1** | In this class, I feel comfortable |
| **Q2** | In this class, I belong to the group |
| **Q3** | I like my class |
|  | **Friendship scale** *(α=.807-.895)* |
| **Q1** | My friend ______ and I hang around and have fun together |
| **Q2** | My friend _____ and I help each other out |
| **Q3** | I am sure I will be friends with _____ for a long time |
| **Q4** | Even if others stopped liking me, _____ would still be my friend |
| **Q5** | My friend _____ and I disagree about many things |
| **Q6** | My friend _____ and I argue with each other |
| **Q7** | My friend _____ and I really care about each other |
| **Q8** | My friend _____ and I get annoyed with each other |
| **Q9** | My friend _____ and I get mad or upset with each other |

|  | **Rejection sensitivity** *(α=.672-.730).* |
| --- | --- |
| **Q1** | Imagine you are the last to leave the class for lunch. As you run down the stairs to the cafeteria, you hear some kids whispering below. You wonder if they are talking about you. |
| **Q1A** | *How nervous would you feel, right then, that those kids were talking bad about you?* |
| **Q1B** | *Do you think they were saying bad things about you?* |
| **Q2** | Imagine you have just moved and you are walking home from school. You wish you had someone to walk home with. You look up and see in front of you another kid from class, and you decide to walk up to this kid and start talking. As you rush to catch up, you wonder if he/she will want to talk to you. |
| **Q2A** | *How NERVOUS would you feel, RIGHT THEN, about whether that kid will want to talk to you?* |
| **Q2B** | *Do you think that kid will want to talk to you?* |
| **Q3** | Imagine you are in class. Everyone is splitting into groups to work on a special project. You sit there and watch lots of other kids get picked. As you wait, you wonder if anyone will want you for their group. |
| **Q3A** | *How nervous would you feel, right then, about whether anyone will choose you?* |
| **Q3B** | *Do you think that anyone will choose you for their group?* |

**Table S2**. Longitudinal associations from Time1 shyness, emotional reactivity, rejection sensitivity to Time 2 loneliness moderated by Time 1 perceived classroom climate.

| **Longitudinal Path** | **β** | **CI [95%]** | ***p*** |
| --- | --- | --- | --- |
| **(Model 1A)** | | | |
| **T1 Emotional reactivity → T2 Loneliness** | .090* | [.021, .158] | .010 |
| **T1 Shyness → T2 Loneliness** | .487** | [.261, .713] | .000 |
| **T1 Classroom climate → T2 Loneliness** | .092 | [-.049, .233] | .202 |
| **T1 Rejection sensitivity → T2 Loneliness** | .118** | [.035, .200] | .005 |
| **T1 Loneliness → T2 Loneliness** | .264** | [.184, .353] | .000 |
| **T1 Shyness x T1 Classroom climate → T2 Loneliness** | -.364** | [-.563, -.166] | .001 |
| **(Model 1B)** | | | |
| **T1 Emotional reactivity → T2 Loneliness** | .506** | [.265, .747] | .000 |
| **T1 Shyness → T2 Loneliness** | .095* | [.010, .181] | .029 |
| **T1 Classroom climate → T2 Loneliness** | .227* | [.014, .440] | .037 |
| **T1 Rejection sensitivity → T2 Loneliness** | .116** | [.034, .198] | .006 |
| **T1 Loneliness → T2 Loneliness** | .271** | [.187, .355] | .000 |
| **T1 Emotional reactivity x T1 Classroom climate → T2 Loneliness** | -.502** | [-.784, -.221] | .011 |
| **(Model 1C)** | | | |
| **T1 Emotional reactivity → T2 Loneliness** | .094** | [.025, .162] | .007 |
| **T1 Shyness → T2 Loneliness** | .102* | [.018, .187] | .018 |
| **T1 Classroom climate → T2 Loneliness** | .053 | [-.058, .164] | .352 |
| **T1 Rejection sensitivity → T2 Loneliness** | .569** | [.350, .788] | .000 |
| **T1 Loneliness → T2 Loneliness** | .253** | [.168, .338] | .000 |
| **T1 Rejection sensitivity x T1 Classroom climate → T2 Loneliness** | -.420** | [-.614, -.226] | .000 |

***Notes.*** *N = 540. Standardized beta weights reported. Models 1 (ABC) describe results from separate models that included interaction terms. Not depicted are cross lagged paths between predictors of loneliness, since they do not change after including interaction terms and are shown in Table 2. Concurrent correlations are given in Table 1. Model fit the data for Model 1A [χ2(18)=24.890, p=.127; TLI=.987; RMSEA =.027 (.000, .050)], Model 1B [χ2(18)=26.350, p=.092; TLI=.984; RMSEA =.029 (.000, .052), and Model 1C [χ2(18)=26.974, p=.080; TLI=.983; RMSEA=.003 (.000, .053)]. Classroom climate = Positive perceived classroom climate.*

**p < .05. **p < .01.*

**Table S3**. Longitudinal associations from Time1 shyness, emotional reactivity, rejection sensitivity to Time 2 loneliness: Results from path analysis of Model 0 before trimming insignificant paths.

| **Longitudinal Path** | **β** | **CI [95%]** | ***p*** |
| --- | --- | --- | --- |
| **Cross lagged paths** | | | |
| **T1 Emotional reactivity → T2 Loneliness** | .120** | [.043, .201] | .003 |
| **T1 Shyness → T2 Loneliness** | .092* | [.005, .179] | .038 |
| **T1 Classroom climate → T2 Loneliness** | -.136** | [-.219, -.052] | .001 |
| **T1 Rejection sensitivity → T2 Loneliness** | .115** | [.031, .199] | .007 |
| **T1 Emotional reactivity → T2 Classroom climate** | -.017 | [-.089, .056] | .653 |
| **T1 Shyness → T2 Classroom climate** | -.0073 | [-.151, .004] | .064 |
| **T1 Rejection sensitivity → T2 Classroom climate** | -.122** | [-.224, -.045] | .002 |
| **T1 Shyness → T2 Emotional reactivity** | .100* | [.017, .183] | .019 |
| **T1 Rejection sensitivity → T2 Emotional reactivity** | .094* | [.010, .177] | .028 |
| **T1 Classroom climate → T2 Emotional reactivity** | -.044** | [-.148, .035] | .272 |
| **T1 Emotional reactivity → T2 Shyness** | .060 | [-.039, .135] | .120 |
| **T1 Rejection sensitivity → T2 Shyness** | .120** | [.040, .200] | .003 |
| **T1 Classroom climate → T2 Shyness** | .029 | [-.071, .105] | .457 |
| **T1 Emotional reactivity → T2 Rejection sensitivity** | .111** | [.012, .187] | .004 |
| **T1 Shyness → T2 Rejection sensitivity** | .0129** | [.048, .209] | .002 |
| **T1 Classroom climate → T2 Rejection sensitivity** | -.042 | [-.119, .034] | .280 |
| **Autoregressive paths** | | | |
| **T1 Loneliness → T2 Loneliness** | .288** | [.200, .367] | .000 |
| **T1 Shyness → T2 Shyness** | .464** | [.446, .613] | .000 |
| **T1 Rejection sensitivity → T2 Rejection sensitivity** | .464** | [.366, .539] | .000 |
| **T1 Emotional reactivity → T2 Emotional reactivity** | .471** | [.400, .542] | .000 |
| **T1 Classroom climate → T2 Classroom climate** | .554** | [.490, .618] | .000 |

***Notes.*** *N = 540. Standardized beta weights reported. Concurrent correlations are given in Table 1. Model fit the data [χ^2^(14)=29.322, p=.009; TLI=.968; RMSEA=.045 (.022, .068)], Classroom climate = Positive perceived classroom climate.*

**p < .05. **p < .01.*
